# NDUFA4L2 promotes glioblastoma progression, is associated with poor survival, and can be effectively targeted by apatinib

**DOI:** 10.1038/s41419-021-03646-3

**Published:** 2021-04-07

**Authors:** Zheng Chen, Xiangyu Wei, Xueyi Wang, Xuan Zheng, Bowen Chang, Lin Shen, Hanshuo Zhu, Min Yang, Shiting Li, Xuesheng Zheng

**Affiliations:** 1 Department of Neurosurgery, XinHua Hospital, Shanghai JiaoTong University School of Medicine, 1665 KongJiang Rd, 200092 Shanghai, China; 2 The Center for Diagnosis and Treatment of Cranial Nerve Diseases of Shanghai JiaoTong University, 200092 Shanghai, China

**Keywords:** CNS cancer, Mitophagy

## Abstract

NADH dehydrogenase [ubiquinone] 1 alpha subcomplex, 4-like 2 (NDUFA4L2) is a subunit of Complex I of the mitochondrial respiratory chain, which is important in metabolic reprogramming and oxidative stress in multiple cancers. However, the biological role and molecular regulation of NDUFA4L2 in glioblastoma (GBM) are poorly understood. Here, we found that NDUFA4L2 was significantly upregulated in GBM; the elevated levels were correlated with reduced patient survival. Gene knockdown of NDUFA4L2 inhibited tumor cell proliferation and enhanced apoptosis, while tumor cells initiated protective mitophagy in vitro and in vivo. We used lentivirus to reduce expression levels of NDUFA4L2 protein in GBM cells exposed to mitophagy blockers, which led to a significant enhancement of tumor cell apoptosis in vitro and inhibited the development of xenografted tumors in vivo. In contrast to other tumor types, NDUFA4L2 expression in GBM may not be directly regulated by hypoxia-inducible factor (HIF)-1α, because HIF-1α inhibitors failed to inhibit NDUFA4L2 in GBM. Apatinib was able to effectively target NDUFA4L2 in GBM, presenting an alternative to the use of lentiviruses, which currently cannot be used in humans. Taken together, our data suggest the use of NDUFA4L2 as a potential therapeutic target in GBM and demonstrate a practical treatment approach.

## Background

Glioblastoma (GBM) is the most common and aggressive malignant primary intracranial tumor of the central nervous system in adult worldwide, and has a poor prognosis. At present, maximum safe resection with postoperative adjuvant ionizing radiotherapy and temozolomide (TMZ) chemotherapy is the standard treatment option for newly diagnosed patients^1^. This standard treatment regimen provides palliative relief for patients with GBM; the median survival time, which has not improved in the past decade, is limited to 14.6 months^2–4^. Thus, an improved understanding of the molecular pathogenesis of GBM and investigation of mechanism-based therapeutic strategies for patients is both essential and urgent.

NADH dehydrogenase [ubiquinone] 1 alpha subcomplex, 4-like 2 (NDUFA4L2), a subunit of Complex I of the mitochondrial respiratory chain, has an important role in metabolic reprogramming and oxidative stress in a variety of malignant tumors^5–7^. In a study of hepatocellular carcinoma, Lai et al. found that NDUFA4L2 was highly upregulated in cancer tissues, compared with normal liver tissues^8^. NDUFA4L2 overexpression was induced by hypoxia and associated with cancer invasion and poor overall survival. NDUFA4L2 was also found to be a direct transcriptional target of hypoxia-inducible factor (HIF)-1α; knockdown of NDUFA4L2 or inhibition of HIF-1α led to marked suppression of tumor growth and metastasis, suggesting that patients with hepatocellular carcinoma patients who exhibit NDUFA4L2 overexpression may be suitable candidates for HIF inhibitor treatment. Similarly, Minton et al.^6^ reported that NDUFA4L2 was induced by HIF-1α in patients with clear cell renal cell carcinoma. NDUFA4L2 was highly expressed in tumor samples, but undetectable in normal kidney tissue samples; moreover, NDUFA4L2 overexpression was correlated with poor overall survival. NDUFA4L2 knockdown-induced autophagy, impaired cell proliferation, and diminished colony formation. Additionally, hypoxia-induced overexpression of NDUFA4L2, which leads to suppression of oxidative phosphorylation, has been observed in lung and colorectal cancer^9,10^. However, it remains unknown whether this phenomenon occurs in GBM. The aim of this study was to explore the expression and biological role of NDUFA4L2 in GBM.

Apatinib, as a multikinase inhibitor, reportedly exhibits promising anti-tumor effects in a variety of tumors^11–13^. In phase III clinical trial, Li et al. found that apatinib was the only drug to effectively treat advanced gastric cancer in patients for whom other chemotherapy regimens failed^14^. Several clinical trials investigating other malignant tumors are currently under investigation, including colorectal cancer, non-small cell lung cancer, esophageal cancer, and breast cancer^15–17^. Although apatinib has been reported to benefit some patients with glioma^18–20^, the underlying mechanism remains unclear.

Here, we found that NDUFA4L2 is significantly upregulated in GBM tissues and associated with poor survival in patients with GBM. NDUFA4L2 knockdown in GBM cells caused cell cycle arrest; it also induced apoptosis and protective mitophagy. Our results indicate that NDUFA4L2 may be a potential target for GBM treatment. Furthermore, our results indicate that apatinib could be a potential therapeutic strategy for GBM by targeting NDUFA4L2 in vivo and in vitro.

## Materials and methods

### Reagents and chemicals, GBM cell culture, and specimens

Reagents and chemicals information was shown in Supplementary Table S1. GBM cell lines T98G, LN229, U87, U251, A172 were purchased and authenticated by the Cell Bank of the Chinese Academy of Sciences (Shanghai, China). SVGp12, U373, U118, U138 were purchased from ATCC. HA1800 was purchased from Sciencell company (Carlsbad, CA, USA). GBM-XX is a primary cell line we isolated and established from the surgical specimens of a patient with World Health Organization grade IV glioblastoma. The culture medium for T98G, LN229, U87, U251, A172, U118, and U138 was composed of DMEM (Life Technologies/Gibco, Carlsbad, CA, USA) and 10% FBS (Life Technologies/Gibco, Carlsbad, CA, USA), 100 U/mL penicillin and 100 μg/mL streptomycin (Gibco, Grand Island, NY, USA). U373 was cultured by RMPI (Life Technologies/Gibco, Carlsbad, CA, USA) + 10% FBS, HA1800 and SVGp12 were cultured by DEME/F12 (Life Technologies/Gibco, Carlsbad, CA, USA) + 10% FBS. These cells were incubated at 37 °C with 5%CO_2_ and 100% humidity. Patients’ glioblastoma multiforme (GBM) tissues and normal brain tissues were obtained from patients who underwent surgery from November 2017 to June 2019 in Xinhua Hospital, and all samples were identified by two independent pathologists. In accordance with the Declaration of Helsinki, prior informed consent was obtained from the patient. This study was approved by the Human Ethics Committee of Xinhua Hospital.

### RNA interference and transfection

Three NDUFA4L2-siRNAs, PINK1-siRNAs, and their negative control (NC) siRNAs (including si-NDUFA4L2^#^1, si-NDUFA4L2^#^2, si-NDUFA4L2^#^3, si-PINK1^#^1, si-PINK1^#^2, si-PINK1^#^3 and si-NC) were purchased from RiboBio, China. And the pcDNA3.1- NDUFA4L2 and the empty vector were purchased from Sangon Biotech, China. The lentivirus vector knockdown NDUFA4L2 (Lv-sh- NDUFA4L2) Stably and lentivirus knockdown Negative Control (sh-NC) were purchased from Cyagen (Guangzhou, China). Stably shRNA-NDUFA4L2-transfected cells were selected by the treatment of puromycin (1 μg/mL, Solarbio, China). The sequences of siRNAs are listed in Supplementary Table S1. Cells were cultured on six-well plates to confluency and transfected with siRNAs or plasmids using Lipofectamine 2000 (Invitrogen, USA) according to the manufacturer’s protocol. After 48 h, cells were harvested for the subsequent experiments. After RNA interference, the group of small interference RNA with the highest knockout efficiency was selected for follow-up experiments.

### Total RNA extraction, reverse transcription, and qPCR

Total RNA was extracted from tissues and cell lines using TRIzol (TaKaRa, Dalian, China). For mRNA analysis, the Primer-Script one-step RT-PCR kit (TaKaRa) was used to reverse transcribe RNA into cDNA. The cDNA templates were amplified by RT-PCR using the SYBR Premix Dimmer Eraser kit (TaKaRa). The primer sequences used were shown in Supplementary Table S2. The relative mRNA expression change was calculated using the 2^−ΔΔCt^ method and normalized with a β-actin housekeeping gene.

### Western blot

Western blot assay was performed as previously described^21^. Briefly, cells were washed with PBS and then lysed with RIPA lysis buffer supplemented with protease inhibitor. Equal amounts of proteins were subjected to SDS-polyacrylamide gel electrophoresis and transferred onto PVDF membranes. Standard western blot was conducted using antibodies as listed in Supplementary Table S3.

### Immunofluorescence analysis

Cells were cultured on glass coverslips in 6-well plates. And after different treatments, cells were fixed in a solution of 4% paraformaldehyde for 45 min. Then permeabilized with 0.1% Triton X-100 for 20 min, and blocked in 5% goat serum for 1.5 h. Coverslips were then incubated in primary antibodies overnight at 4 °C in the dark. Then cells were washed and incubated in secondary antibody (Abcam) for 1.5 h at 37 °C. The slips were immediately analyzed using fluorescence microscopy (Olympus BX51, Japan).

### Cell proliferation and cell cycle analysis

Cell proliferation was assessed by CCK8 (Dojindo, Japan), Ethynyldeoxyuridine (EdU) retention assay, and colony formation assays. For the CCK8 assay, 1.5 × 10^3^ cells/well cells were seeded into 96-well plates and the absorbance (450 nm) was measured every 24 h for 96 h. For Ethynyldeoxyuridine (EdU) retention assay, the EdU Cell Proliferation kit (Beyotime, China) was applied. 48 h after transfection, approximately 5 × 10^3^ transfected cells were seeded into each well of 96-well plates, 10 μM EdU labeling media was added and incubated for 2 h in a CO_2_ incubator at 37 °C. Then cells were treated with 4% paraformaldehyde, 0.5% Triton X-100, and anti-EdU working solution successively. The percentage of EdU positive cells was calculated using fluorescent microscopy. For colony formation assay, 1 × 10^3^ cells were seeded into every well of 6-well plates and cultured in media with 10% FBS. Two weeks later, cells were treated with 4% paraformaldehyde and stained with 0.1% crystal violet. The number of visible colonies was counted.

### Flow cytometry

Cell apoptosis was measured by flow cytometry with an annexin V-FACS apoptosis detection kit (BD Biosciences, USA) as previously described^21^. The cell cycle was analyzed by flow cytometry with a FACS Calibur (BD Biosciences, USA). Cells were collected and fixed by pre-cold 75% ethanol at 4 °C overnight and incubated in staining solution (10 μg/mL propidium iodide +5 U/mL RNaseA) for 30 min at 37 °C. The flow cytometer was used for evaluation.

### Stably expressing stubRFP-sensGFP-LC3

We purchased the adenovirus vector containing the stubRFP-sensGFP-LC3 reporter from Hanbio Biotechnology (Shanghai, China). 1 μg/mL puromycin was used for selecting Stably expressing stubRFP-sensGFP-LC3 cells. After different treatment, the cells were fixed and then examined using fluorescence microscopy (Olympus, Japan).

### Transmission electron microscopy (TEM)

After different treatment, Cells were fixed for 2 h using 2% glutaraldehyde containing 0.1 mol/L phosphate-buffered saline at 4 °C, incubated at 4 °C by 1% osmium tetroxide containing 0.1 mol/L phosphate-buffered saline for 2 h, dehydrated in graded ethanol, saturated in graded Epikote and embedded, cut into 50 nm ultrathin sections, stained with lead citrate and finally viewed using Philip CM-120 TEM (Philips, Netherlands).

### Animal xenografts

A tumor xenograft experiment was performed to explore the function of NDUFA4L2 in vivo. LN229 cells (100 μL, ~5.0 × 10^6^ cells) stably transfected with Lv-shRNA-NDUFA4L2 or Lv-shRNA-NC were subcutaneously injected into 4-week-old male nude mice. Mice were divided into four groups (*n* = 5 per group): Lv-shRNA-NC, Lv-shRNA-NC + Mdivi-1, Lv-shRNA-NDUFA4L2, and Lv-shRNA-NDUFA4L2 + Mdivi-1. A concentration of 3 mg/kg Mdivi-1 was chosen for in vivo experiments^22^. Seven days after subcutaneous injection, mice in the Lv-shRNA-NC + Mdivi-1 and Lv-shRNA-NDUFA4L2 + Mdivi-1 groups received an intraperitoneal injection of Mdivi-1 dissolved in corn oil at 2-day intervals for 4 weeks; the Lv-shRNA-NC and Lv-shRNA-NDUFA4L2 groups received an equal volume of corn oil at 2-day intervals for 4 weeks as a control. Tumor volumes were calculated as 0.5 × length × width^2^ on a weekly basis. After 4 weeks, mice were euthanized, and tumors were then excised and weighed; tumor tissues were used for immunohistochemical analysis.

The inhibitory effect of apatinib in GBM cells was verified by targeting NDUFA4L2 in vivo. Seven days after mice had been subcutaneously injected with LN229 cells (100 μL, ~5.0 × 10^6^ cells), mice were randomly divided into four groups (*n* = 15 per group): control, Mdivi-1 (3 mg/kg), apatinib (50 mg/kg), and apatinib (50 mg/kg) + Mdivi-1 (3 mg/kg)^12,23^. Seven mice from each group were then randomly selected to receive different drug treatments at 2-day intervals for 4 weeks. The experiment was then performed as described above. The remaining eight mice in each group received drug treatment at 2-day intervals until the mice died. The survival time of the mice was recorded, and median survival rates were calculated.

### Statistical analysis

All statistical analyses were performed using SPSS Statistics, version 20.0 (IBM Corp., Armonk, NY, USA). Data are expressed as means ± standard deviations. Graphs were generated using GraphPad Prism, version 5.0.1 (GraphPad, La Jolla, CA, USA). For relative data analysis, the mean value of the control group was defined as 1% or 100%. Differences between the two groups were compared using the unpaired Student’s *t*-test. Differences between multiple groups were compared with a one-way analysis of variance. The Kaplan–Meier method was used to assess survival, and log-rank tests were used to determine significance. All tests were two-tailed and a *p*-value < 0.05 was considered statistically significant.

## Results

### NDUFA4L2 mRNA and protein levels are elevated in human GBM tissues and associated with short survival time in patients with GBM

**Fig. 1:**
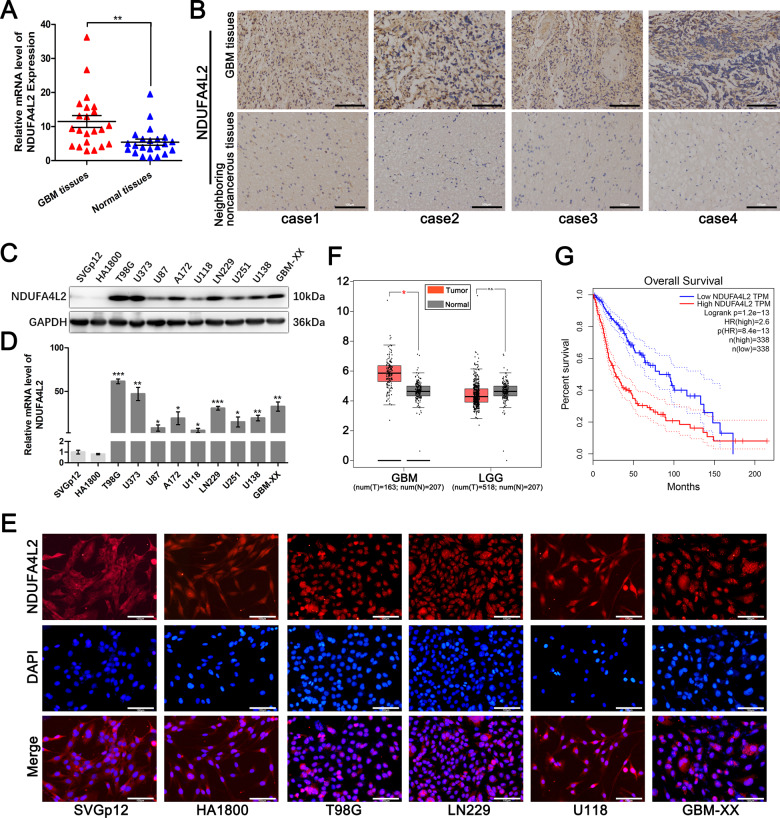
NDUFA4L2 expression is elevated in glioblastoma (GBM) and is clinically significant. **A** Relative expression of NDUFA4L2 in GBM (*n* = 22) and normal brain (*n* = 22) tissues was detected by qRT-PCR. Relative expression of NDUFA4L2 was normalized to ACTIN. **B** NDUFA4L2 expression in GBM and neighboring non-cancerous tissues was determined by immunohistochemical staining. **C**, **D** Expression levels of NDUFA4L2 in GBM cell lines (T98G, U373, U87, A172, U118, LN229, U251, U138, and GBM-XX) and normal human brain astroglia cell lines SVGp12 and HA1800 were determined by western blotting and qRT-PCR. Relative expression levels of NDUFA4L2 were normalized to ACTIN in qRT-PCR. **E** NDUFA4L2 expression levels in GBM and normal human brain astroglia cell lines were measured by immunofluorescence assay. **F** Relative expression levels of NDUFA4L2 in clinical GBM (*n* = 163) and normal brain (*n* = 207) tissues were analyzed in TCGA and GTEx data. **G** Based on TCGA data, the Kaplan–Meier method with log-rank test was used to analyze the overall survival curves of patients with glioma in high and low NDUFA4L2 expression groups (*n* = 338, log-rank = 2.6, *p* < 0.001). Means ± standard deviations (SDs) of triplicate experiments are plotted. *p < 0.05, **p < 0.01, ***p < 0.001, n.s. not statistically significant.

To identify the role of NDUFA4L2 in GBM, we first measured mRNA levels of NDUFA4L2 in 22 GBM and 22 normal brain tissue samples by qRT-PCR (Fig. 1A). The transcript levels of NDUFA4L2 were significantly higher in GBM tissues than in normal brain tissues. We randomly chose four pairs of GBM tissues and corresponding adjacent non-cancerous tissues from patients; these tissues were used for immunohistochemical staining to measure protein levels of NDUFA4L2. The results showed that NDUFA4L2 was observably elevated in GBM tissues (Fig. 1B). Next, we measured NDUFA4L2 mRNA and protein levels in nine human GBM cell lines and two normal brain astroglia cell lines (SVGp12 and HA1800). The mRNA and protein expression levels of NDUFA4L2 were generally higher in the GBM cell lines than in SVGp12 and HA1800 cells (Fig. 1C, D). We then selected T98G, LN229, U118, and GBM-XX cells for immunofluorescence assays. The results of the immunofluorescence assays were consistent with the qRT-PCR and western blot assays, indicating that NDUFA4L2 levels were generally higher in GBM cell lines than in SVGp12 and HA1800 cells (Fig. 1E).

To further verify the correlations between protein levels of NDUFA4L2 and clinical characteristics of patients with glioma, we analyzed NDUFA4L2 expression levels in TCGA and GTEx data. As shown in Fig. 1F, there was no significant difference between the low-grade glioma and normal brain tissue groups in terms of NDUFA4L2 expression level; however, NDUFA4L2 was highly elevated in GBM tissues, compared to normal brain tissues. Furthermore, Kaplan–Meier analysis indicated that patients with higher NDUFA4L2 expression levels have a shorter survival time than patients with lower NDUFA4L2 expression levels (Fig. 1G). Collectively, these data demonstrate that NDUFA4L2 mRNA and protein levels are elevated in human GBM cells and associated with shorter survival time in patients with GBM.

### NDUFA4L2 promotes GBM cell proliferation in vitro

**Fig. 2:**
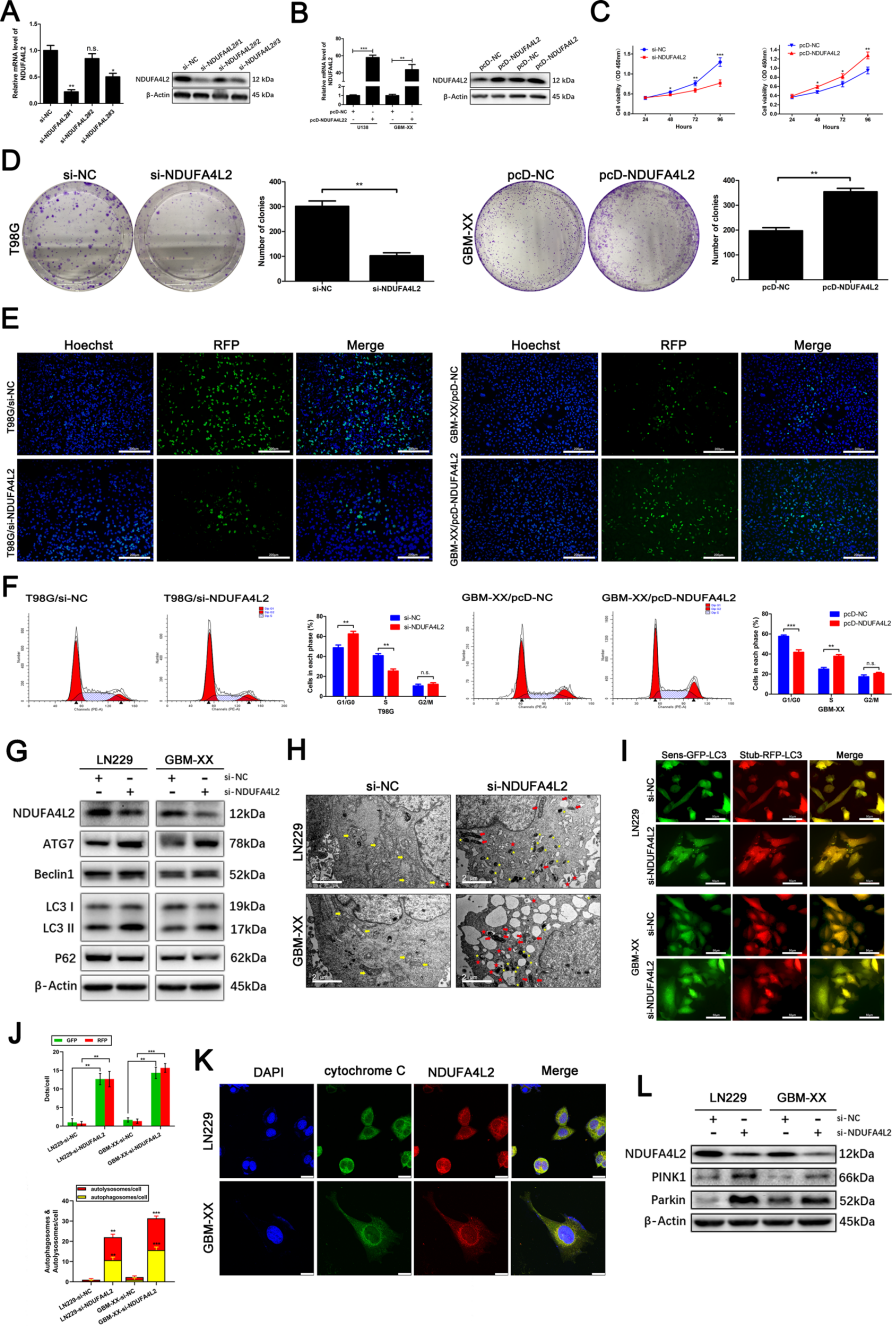
Effect of NDUFA4L2 on GBM cell growth and NDUFA4L2 knockdown induces mitophagy in GBM cells. **A** mRNA and protein levels of NDUFA4L2 in T98G cells transfected with three different small interfering RNAs (siRNAs) against NDUFA4L2 or si-NC were quantified by qRT-PCR and western blotting. **B** qRT-PCR and western blotting results of U138 and GBM-XX cells transfected with pcDNA-NC or pcDNA-NDUFA4L2 are shown. **C** Cell viabilities of si-NDUFA4L2-transfected T98G and pcDNA-NDUFA4L2-transfected LN229 cells were determined by CCK8 assay. **D** Colony-forming abilities of si-NDUFA4L2-transfected T98G and pcDNA-NDUFA4L2-transfected GBM-XX cells were determined by colony formation assays. **E** Cell viabilities of si-NDUFA4L2-transfected T98G and pcDNA-NDUFA4L2-transfected GBM-XX cells were determined by 5-ethynyl-2′-deoxyuridine retention assay. **F** Flow cytometry was performed to analyze cell cycle progression in si-NDUFA4L2-transfected T98G and pcDNA-NDUFA4L2-transfected GBM-XX cells. **G** Protein levels of LC3, p62, Beclin-1, and ATG7 in LN229 and GBM-XX cells transfected with si-NDUFA4L2 or si-NC were determined by western blotting. **H** Representative transmission electron microscopy images of si-NDUFA4L2-transfected LN229 and GBM-XX cells. **I**, **J** si-NDUFA4L2 or si-NC transfected LN229 and GBM-XX cells stably expressing the stubRFP-sensGFP-LC3 fusion protein were established and observed by fluorescence microscopy. **K** Immunofluorescence images showing DAPI (blue), cytochrome C (green), and NDUFA4L2 (red) staining, as well as merged images of the three signals. **L** Protein levels of PINK1 and Parkin in LN229 and GBM-XX cells transfected with si-NDUFA4L2 or si-NC were determined by western blotting. The images shown are representative of three experiments. Means ± SDs of triplicate experiments are plotted. *p < 0.05, **p < 0.01, ***p < 0.001, n.s. not statistically significant.

To explore whether NDUFA4L2 is involved in GBM cell proliferation, we selected si-NDUFA4L2^#^1 from three siRNAs for subsequent experiments because of its higher efficiency (Fig. 2A). We also established stable NDUFA4L2-overexpressing U138 and GBM-XX cells via transfection of pcDNA-NDUFA4L2 (Fig. 2B). Cell Counting Kit-8 (CCK8) and colony formation assays were performed to evaluate the role of NDUFA4L2 in GBM cell proliferation in vitro. As shown in Fig. 2C, D, NDUFA4L2 knockdown significantly inhibited T98G cell proliferation, while NDUFA4L2 overexpression significantly promoted GBM-XX cell proliferation. Furthermore, a 5-ethynyl-2′-deoxyuridine retention assay confirmed the inhibitory effect of NDUFA4L2 knockdown on T98G cell proliferation; NDUFA4L2 overexpression significantly promoted GBM-XX cell proliferation (Fig. 2E). To investigate whether the effect of NDUFA4L2 on GBM cell proliferation in vitro was due to cell cycle regulation, we performed flow cytometry. NDUFA4L2 knockdown led to G1 phase arrest of T98G cells, while NDUFA4L2 overexpression promoted GBM-XX cell cycle progression (Fig. 2F). Together, these results indicate that NDUFA4L2 regulates the G1/S cell cycle transition in GBM cells.

### NDUFA4L2 knockdown induces apoptosis and initiates protective mitophagy in GBM

**Fig. 3:**
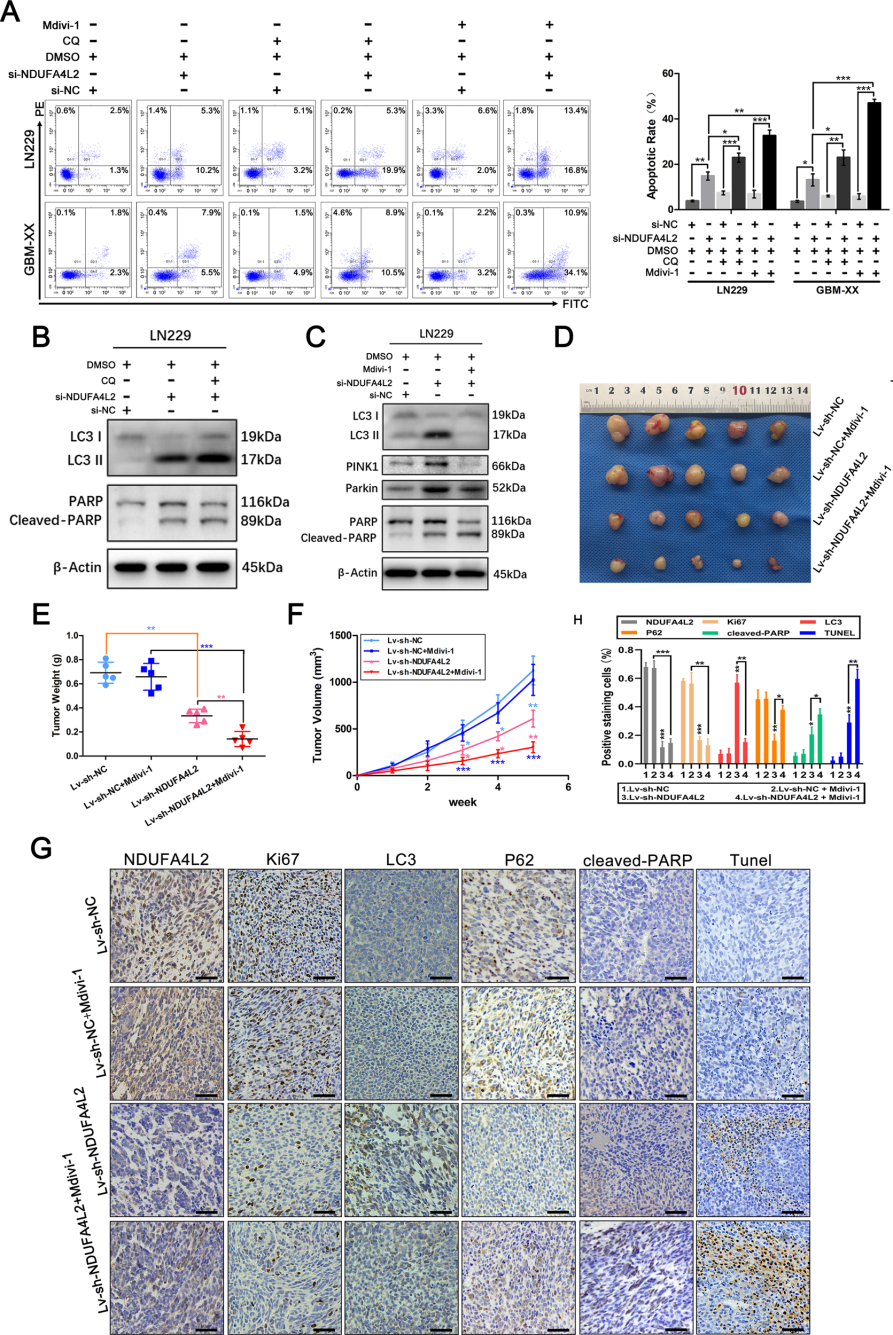
NDUFA4L2 knockdown induces apoptosis, which is further enhanced by autophagy inhibitor treatment in vitro and vivo. **A** Apoptosis rates in LN229 and GBM-XX cells treated with chloroquine (CQ; 10 μM) or Mdivi-1 (5 μM) were determined by flow cytometry (*n* = 3). **B** Protein levels of LC3, PARP, and cleaved-PARP in LN229 cells, following treatment with CQ (10 μM), were determined by western blotting. **C** Protein levels of LC3, PINK1, Parkin, PARP, and cleaved-PARP in LN229 cells, following treatment with Mdivi-1 (5 μM), were determined by western blotting. **D**–**F** Nude mice carrying tumors from LN229-transfected (sh-NDUFA4L2 or sh-NC by lentivirus) cells untreated or treated with Mdivi-1 (3 mg/kg) are shown (*n* = 5). The average tumor weight for each group was calculated. Tumor growth curves were calculated for each week. **G-H** NDUFA4L2, Ki67, LC3, p62, and cleaved-PARP expression levels, as well as positive cell numbers, were determined by immunohistochemical staining; apoptosis was assessed by TUNEL assay. Scale bar = 50 μm. Means ± SDs of triplicate experiments are plotted. *p < 0.05, **p < 0.01, ***p < 0.001.

We also found that when we knocked down the expression level of NDUFA4L2 in LN229 and GBM-XX cells, apoptosis of these cells increased slightly. Flow cytometry analysis revealed that, compared with the control (LN229 and GBM-XX cells transfected with si-NC), NDUFA4L2 knockdown-induced enhanced apoptosis (Fig. 3A). Immunoblot analysis revealed that the conversion of LC3-I to LC3-II was considerably elevated, along with the expression levels of Beclin-1 and ATG7; moreover, the expression of p62 was markedly inhibited in si-NDUFA4L2-transfected LN229 and GBM-XX cells (Fig. 2G). These results suggest that NDUFA4L2 knockdown might induce cell apoptosis and initiate autophagy in GBM cells. To further validate whether autophagic flux was enhanced in NDUFA4L2 knockdown cells, we used adenovirus to establish stably expressing stubRFP-sensGFP-LC3 GBM cells; we used these cells to localize and assess autophagic flux. SensGFP is sensitive to the change in pH caused by the fusion of autophagosomes and lysosomes, while stubRFP is stable. Fluorescence microscopy revealed elevated autophagosomal-lysosomal fusion in si-NDUFA4L2-transfected LN229 and GBM-XX cells (Fig. 2I, J). Moreover, we analyzed the effects of si-NC treatment and NDUFA4L2 knockdown in LN229 and GBM-XX cells by transmission electron microscopy (TEM) (Fig. 2H). Compared with si-NC-treated cells, the volumes of mitochondria in NDUFA4L2 knockdown LN229 and GBM-XX cells were varied, the color of the mitochondrial matrix was darker, and the mitochondrial cristae were disordered; some mitochondria were fragmented and vacuolized. TEM images showed that NDUFA4L2 knockdown cells had a higher number of autophagosomes/autolysosomes than si-NC-treated cells (yellow arrows indicate normal morphology of mitochondria in GBM cells, red arrows indicate abnormal morphology of mitochondria in NDUFA4L2 knockdown cells; red stars indicate autophagosomes, and yellow stars indicate autolysosomes). These results indicate abnormal morphology of mitochondria in NDUFA4L2 knockdown cells, suggesting that NDUFA4L2 knockdown may induce mitophagy. Subsequently, we performed co-immunofluorescence analysis to determine the location of NDUFA4L2 in GBM cells. NDUFA4L2 colocalized with cytochrome C in LN229 and GBM-XX cells, suggesting that NDUFA4L2 is localized to mitochondria in GBM cells (Fig. 2K). PINK1/Parkin has an important role in cell mitophagy; accumulation of PINK1 protein in mitochondria recruits Parkin and activates mitophagy^24–26^. Therefore, we performed immunoblot analysis to detect PINK1 and Parkin protein levels (Fig. 2L); PINK1 and Parkin protein levels were markedly elevated by NDUFA4L2 knockdown in LN229 and GBM-XX cells. Additionally, we carried out immunofluorescence staining assays to check for PINK1 and PARKIN translocation on mitochondria (Supplementary Fig. S1), analyzed mitochondrial marker levels including COXII, COXIV, TOM20, TIM23 by western blot analysis (Supplementary Fig. S2), and evaluated the mitochondrial mass by mitotracker probe (Supplementary Fig. S3). Collectively, these results suggest that NDUFA4L2 knockdown induces cell apoptosis and initiates mitophagy in GBM.

To further explore the relationship between apoptosis and mitophagy induced by NDUFA4L2 knockdown, we performed flow cytometry to assess apoptosis of si-NC-treated and NDUFA4L2 knockdown cells in the presence or absence of chloroquine (CQ) and Mdivi-1. CQ and Mdivi-1 are two mitophagy inhibitors; CQ raises the intralysosomal pH and suppresses the fusion of autophagosomes and lysosomes at a late stage, while Mdivi-1 inactivates PINK1/Parkin and blocks autophagosome formation in mitophagy at an early stage^27–29^. As shown in Fig. 3A, compared with the control group, cells pretreated with CQ and Mdivi-1 were more sensitive to NDUFA4L2 knockdown, and the apoptotic rates were significantly elevated. Mdivi-1 treatment increased the rate of apoptosis to a greater extent than CQ. Additionally, western blotting of LN229 cells showed that the combination of CQ and si-NDUFA4L2 elevated the expression levels of LC3-II and cleaved-PARP in LN229 cells, compared with only si-NDUFA4L2 treatment (Fig. 3B). Treatment of LN229 cells with Mdivi-1 and si-NDUFA4L2 reversed the elevated expression levels of PINK1, Parkin, and LC3-II induced by si-NDUFA4L2 alone; it also efficiently elevated the levels of cleaved-PARP (Fig. 3C). Taken together, these results suggest that NDUFA4L2 knockdown induces apoptosis and initiates protective mitophagy in GBM.

### Tumor suppression induced by NDUFA4L2 knockdown is enhanced by Mdivi-1 in vivo

To explore the function of NDUFA4L2 in GBM cells in vivo, LN229 cells stably transfected with Lv-shRNA-NDUFA4L2 or Lv-sh-NC were subcutaneously injected into nude mice. As described previously, 3 mg/kg Mdivi-1 was used for in vivo experiments^28,30^. Tumors developed significantly more rapidly in the Lv-sh-NC group than in the Lv-shRNA-NDUFA4L2 group; after 4 weeks, there was no statistically significant difference between the Lv-sh-NC and LV-sh-NC + Mdivi-1 groups (Fig. 3D, F). However, compared to Lv-shRNA-NDUFA4L2, the Lv-shRNA-NDUFA4L2 + Mdivi-1 treatment group showed more efficient suppression of subcutaneous tumor growth. The results regarding tumor weight were consistent with the results regarding tumor volume (Fig. 3E). Furthermore, immunohistochemical staining of NDUFA4L2, Ki67, LC3, p62, and cleaved-PARP showed that compared to the Lv-shRNA-NC group, NDUFA4L2 and Ki67 were markedly downregulated in the Lv-shRNA-NDUFA4L2 group, while the expression level of cleaved-PARP was significantly upregulated. Immunohistochemical staining also confirmed that NDUFA4L2 knockdown resulted in elevated mitophagic activity in GBM cells in vivo (Fig. 3G, H). Compared with the Lv-shRNA-NDUFA4L2 and Lv-shRNA-NDUFA4L2 + Mdivi-1 groups, mitophagic activity in GBM cells was markedly reduced in vivo, and the levels of cleaved-PARP were significantly elevated. These results were further confirmed by the TUNEL assay, which showed that inhibition of mitophagy could enhance the effect of NDUFA4L2 knockdown-induced growth inhibition in GBM cells in vivo, consistent with the aforementioned in vitro results.

### NDUFA4L2 may not be directly regulated by HIF-1α in GBM

**Fig. 4:**
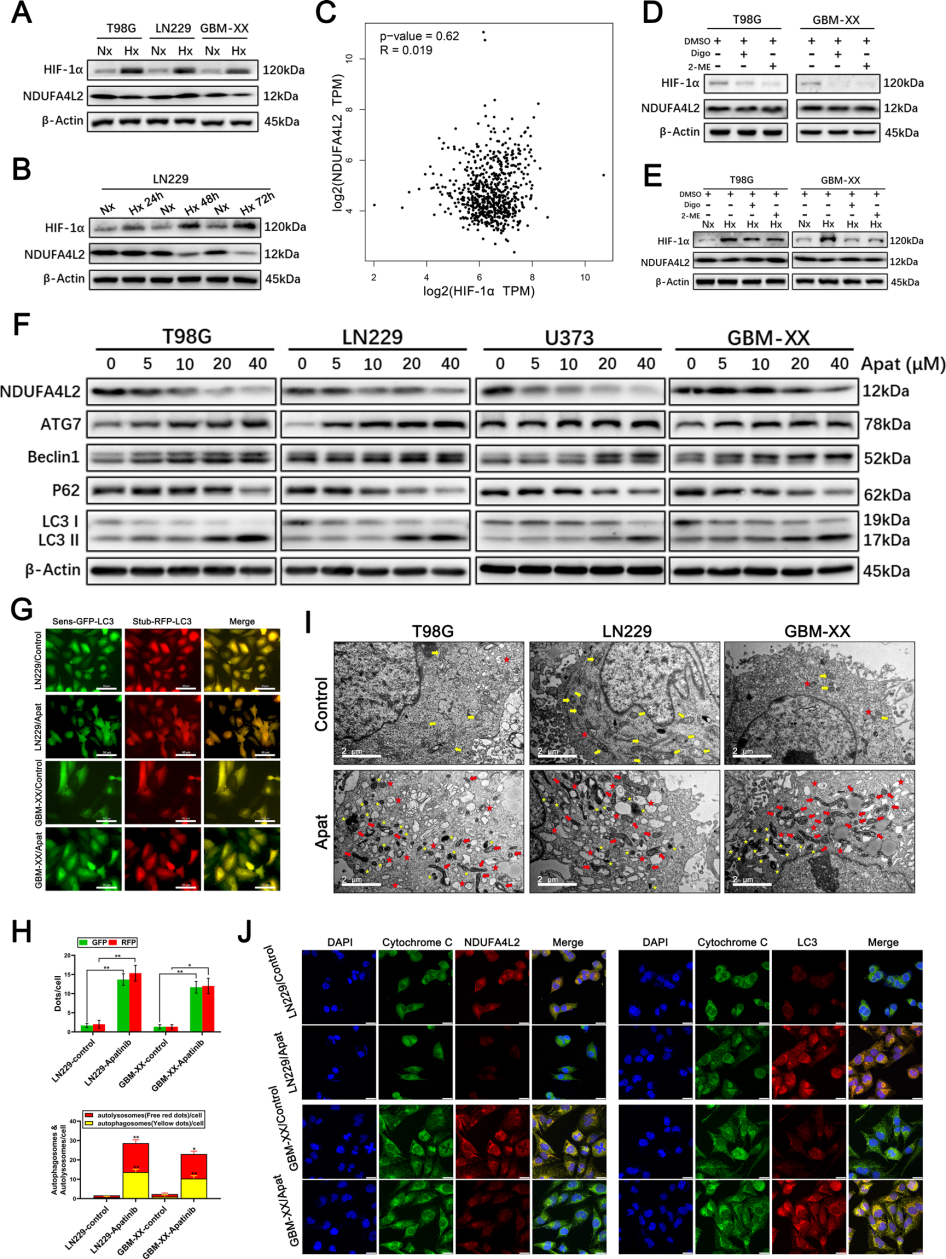
NDUFA4L2 is not regulated by HIF-1α in GBM and Apatinib effectively inhibits NDUFA4L2 expression and induces autophagy in GBM cells. **A** Immunoblot analysis of HIF-1α and NDUFA4L2 protein levels in T98G, LN229, and GBM-XX cells cultured under normoxic or hypoxic conditions (1% O_2_ for cell cultures). **B** Immunoblot analysis of HIF-1α and NDUFA4L2 protein levels in LN229 cells cultured under normoxia or hypoxia for 24, 48, and 72 h. **C** Based on TCGA data, the correlation between the expression levels of HIF-1α and NDUFA4L2 in patients with glioma was analyzed using the Pearson method. **D** Protein levels of HIF-1α and NDUFA4L2 in T98G and GBM-XX cells cultured under normoxia with digoxin (100 nM) or 2-ME (10 μM) were determined by western blotting. **E** Protein levels of HIF-1α and NDUFA4L2 in T98G and GBM-XX cells cultured under hypoxia (24 h) with digoxin or 2-ME were determined by western blotting. The images shown are representative of three experiments. **F** Immunoblot analysis of NDUFA4L2, LC3, p62, Beclin-1, and ATG7 protein levels in T98G, LN229, U373, and GBM-XX cells following treatment with apatinib (0, 5, 10, 20, and 40 μM). **G**, **H** Apatinib-treated LN229 and GBM-XX cells that stably express the stubRFP-sensGFP-LC3 fusion protein were observed by fluorescence microscopy. **I** Representative transmission electron microscopy images of T98G, LN229, and GBM-XX cells following treatment with apatinib (20 μM). **J** Immunofluorescence images of apatinib-treated LN229 or GBM-XX cells showing DAPI (blue), cytochrome C (green), and NDUFA4L2 or LC3 (red) staining, as well as merged images of the three signals. The images shown are representative of three experiments.

Several previous studies of human malignant neoplasms have revealed that HIF-1α has a role in the direct regulation of NDUFA4L2 in cancer cells^6,8^. To explore whether HIF-1α regulates NDUFA4L2 in GBM cells, we performed western blotting to examine the expression levels of HIF-1α and NDUFA4L2 under hypoxic conditions. Notably, the results showed that compared with cells in normoxic conditions, the protein levels of HIF-1α in T98G, LN229, and GBM-XX cells were significantly elevated under hypoxia (for 24 h); however, there were no consistent changes in the expression of NDUFA4L2 (Fig. 4A). Using LN229 cells, we measured changes in the expression levels of HIF-1α and NDUFA4L2 after 24, 48, and 72 h of hypoxia. As shown in Fig. 4B, the expression of HIF-1α showed time-dependent enhancement under hypoxic conditions, while NDUFA4L2 levels did not increase consistently. To further validate the effect of HIF-1α on NDUFA4L2 levels in GBM, we analyzed the correlation between the expression levels of HIF-1α and NDUFA4L2 in GBM tissues of clinical patients in The Cancer Genome Atlas (TCGA) database. There was no significant correlation between the expression of HIF-1α and NDUFA4L2 in clinical GBM tissues (Fig. 4C). Additionally, we used the HIF-1α inhibitors digoxin and 2-methoxyestradiol (2-ME) to assess the inhibitory effect of NDUFA4L2 under normoxic and hypoxic conditions in GBM cells. Treatment with digoxin (100 nM) or 2-ME (10 μM) significantly reduced the expression levels of HIF-1α in T98G and GBM-XX cells in both normoxia (Fig. 4D) and hypoxia conditions (Fig. 4E) but did not inhibit the expression of NDUFA4L2. Collectively, these data suggest that NDUFA4L2 is not directly regulated by HIF-1α in GBM cells and that HIF-1α inhibitors do not inhibit NDUFA4L2 expression.

### Apatinib induces protective mitophagy, arrests the cell cycle, and induces apoptosis by targeting NDUFA4L2 in GBM

NDUFA4L2 may be a promising therapeutic target for the treatment of GBM; however, HIF-1α inhibitors do not directly inhibit NDUFA4L2 in GBM. We found that apatinib shows promising clinical potential in the treatment of patients with GBM by targeting NDUFA4L2. Western blotting using T98G, LN229, U373, and GBM-XX cell lines revealed that apatinib (5, 10, 20, and 40 μM) effectively inhibited the expression of NDUFA4L2 in a dose-dependent manner (Fig. 4F). With a gradual reduction in NDUFA4L2, the conversion of LC3-I to LC3-II and the expression levels of Beclin-1 and ATG7 were elevated; in contrast, the expression of p62 was downregulated. Fluorescence microscopy of LN229 and GBM-XX cells revealed enhanced autophagosomal-lysosomal fusion in apatinib-treated groups (Fig. 4G, H). TEM also revealed abnormal mitochondrial morphology and elevated numbers of autophagosomes/autolysosomes in apatinib-treated T98G, LN229, and GBM-XX cells (Fig. 4I). Subsequently, we performed co-immunofluorescence analysis using LN229 and GBM-XX cells. We found that the expression of NDUFA4L2 was markedly downregulated, while the expression of LC3 was markedly elevated in apatinib-treated groups. Moreover, colocalization of NDUFA4L2 and cytochrome *C*, as well as colocalization of LC3 and cytochrome *C* in mitochondria, was evident in apatinib-treated GBM cells (Fig. 4J).

**Fig. 5:**
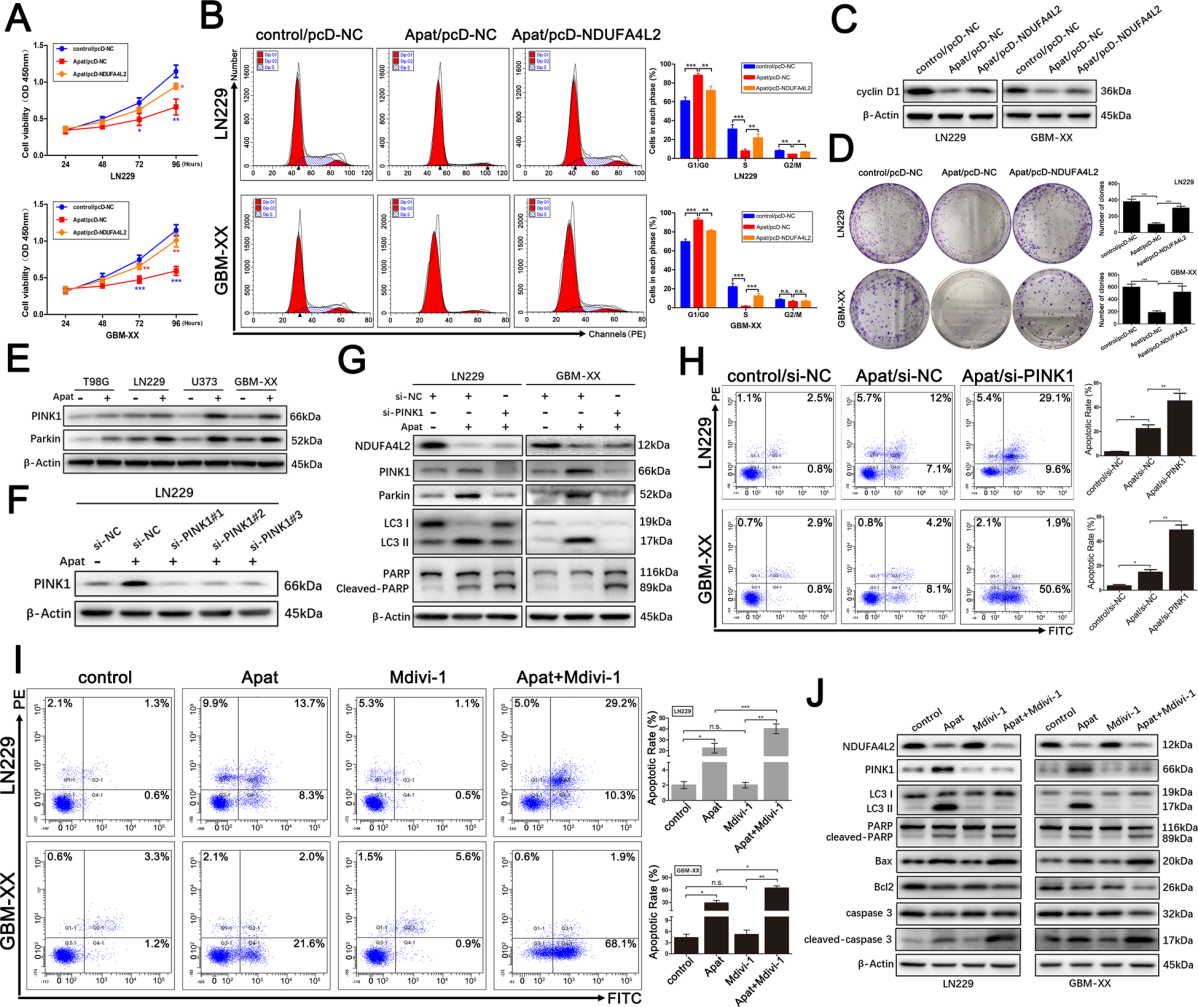
Apatinib induces cell cycle arrest by targeting NDUFA4L2 and induces apoptosis of GBM cells, which is further enhanced by PINK1 knockdown or treatment with mitophagy inhibitor. **A** Cell viabilities of pcDNA-NDUFA4L2 or pcDNA-NC-transfected LN229 and GBM-XX cells treated with apatinib (20 μM) were determined by CCK8 assay. **B** Cell cycle progression in pcDNA-NDUFA4L2 or pcDNA-NC-transfected LN229 and GBM-XX cells treated with apatinib (20 μM) were determined by flow cytometry. **C** Protein levels of cyclin D1 in pcDNA-NDUFA4L2 or pcDNA-NC-transfected LN229 and GBM-XX cells treated with apatinib (20 μM) were determined by western blotting. **D** Colony formation abilities of pcDNA-NDUFA4L2 or pcDNA-NC-transfected LN229 and GBM-XX cells treated with apatinib (20 μM) were determined by colony formation assay. **E** Immunoblot analysis of PINK1 and Parkin protein levels in T98G, LN229, U373, and GBM-XX cells treated with apatinib (20 μM). **F** Protein levels of PINK1 in LN229 cells were determined by western blotting. **G** Protein levels of NDUFA4L2, PINK1, Parkin, LC3, PARP, and cleaved-PARP in si-PINK1 or si-NC-transfected LN229 and GBM-XX cells with apatinib (20 μM) were determined by western blotting. **H** Apoptosis rates in si-PINK1 or si-NC-transfected LN229 and GBM-XX cells with apatinib (20 μM) were determined by flow cytometry (*n* = 3). **I** Apoptosis rates in LN229 and GBM-XX cells treated with apatinib (20 μM), Mdivi-1 (5 μM), or apatinib + Mdivi-1 was determined by flow cytometry (*n* = 3). **J** Immunoblot analysis of NDUFA4L2, PINK1, LC3, PARP, cleaved-PARP, Bax, Bcl2, caspase 3, and cleaved-caspase 3 protein levels in LN229 and GBM-XX cells treated with apatinib (20 μM), Mdivi-1 (5 μM), or apatinib + Mdivi-1. Means ± SDs of triplicate experiments are plotted. *p < 0.05, **p < 0.01, ***p < 0.001, n.s. not statistically significant.

To validate whether apatinib is involved in the arrest of GBM cell proliferation by targeting NDUFA4L2, we performed CCK8 assays in LN229 and GBM-XX cells. As shown in Fig. 5A, cell viability was significantly inhibited by treatment with apatinib; however, the reduction in cell viability was effectively reversed by pcDNA-NDUFA4L2-mediated overexpression of NDUFA4L2. Flow cytometry suggested that apatinib was involved in the inhibition of G1/S cell cycle transition in GBM cells (Fig. 5B). Cyclin D1 is an important marker of G1 phase arrest in the cell cycle^31^. Western blotting revealed that the expression level of cyclin D1 was downregulated following treatment with apatinib; this was effectively reversed by overexpression of NDUFA4L2 (Fig. 5C). Additionally, colony formation assay results were consistent with the results of the CCK8 assay, flow cytometry, and western blotting (Fig. 5D).

We next explored whether apatinib upregulates PINK1 and activates PINK1/Parkin-mediated mitophagy by targeting NDUFA4L2 in GBM. PINK1 and Parkin levels in the GBM cell lines were significantly elevated after treatment with apatinib (Fig. 5E). Next, we designed three PINK1 siRNAs to transfect apatinib-treated LN229 cells; si-PINK1^#^1 was selected for subsequent experiments because of its higher efficiency (Fig. 5F). Western blotting revealed that, compared with cells treated with apatinib alone, apatinib-treated cells with inhibited PINK1 expression exhibited a significant reduction in autophagy activity, along with elevated levels of the apoptosis-related protein cleaved-PARP (Fig. 5G). Additionally, flow cytometry revealed rates of apoptosis consistent with those of western blotting (Fig. 5H). To further verify the above results, we determined the effects of treatment with the mitophagy inhibitor Mdivi-1. Flow cytometry and western blotting revealed similar results (Fig. 5I, J). Taken together, these data indicate that apatinib arrests the cell cycle, induces apoptosis, and initiates protective mitophagy by targeting NDUFA4L2 in GBM cells.

### Tumor suppression induced by apatinib targeting of NDUFA4L2 is enhanced by Mdivi-1 in vivo

**Fig. 6:**
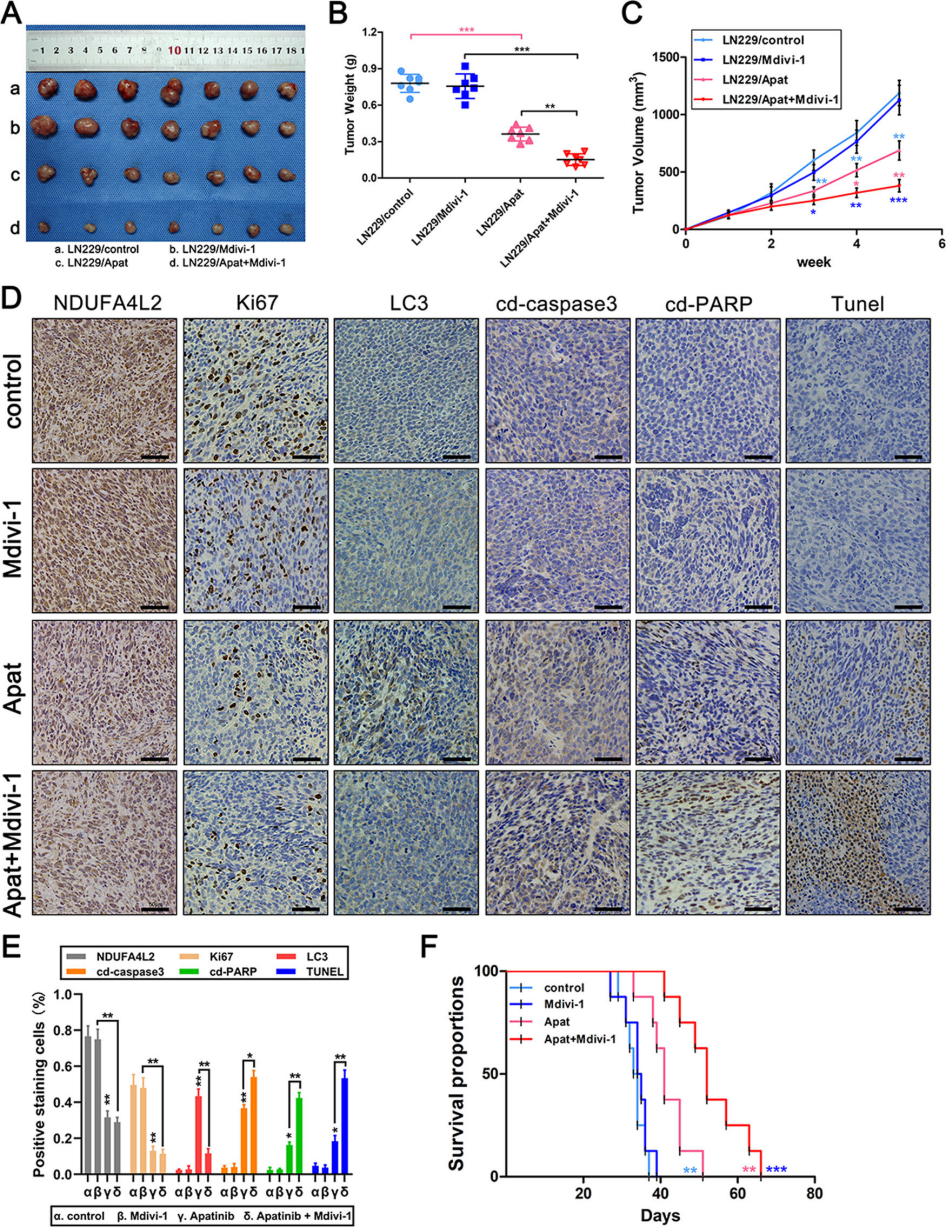
Tumor suppression induced by apatinib targeting of NDUFA4L2 is enhanced by Mdivi-1 in vivo. **A**–**C** Nude mice carrying tumors from LN229 cells treated with apatinib (50 mg/kg), Mdivi-1 (3 mg/kg), or apatinib + Mdivi-1 are shown (*n* = 7). Average tumor weights were calculated for all groups. Tumor growth curves were calculated for each week. **D**, **E** NDUFA4L2, Ki67, LC3, cleaved-caspase 3, and cleaved-PARP expression levels, as well as positive cell numbers, were determined by immunohistochemical staining; apoptosis was assessed by TUNEL assay. Scale bar = 50 μm. **F** Tumor xenografts were established by subcutaneous inoculation of LN229 cells into the armpit of the right upper limb of nude mice. Survival rates of the four groups (control, Mdivi-1, apatinib, and apatinib + Mdivi-1) of nude mice are represented in a Kaplan–Meier plot (*n* = 8). Means ± SDs of triplicate experiments are plotted. *p < 0.05, **p < 0.01, ***p < 0.001.

To further examine the inhibitory effect of apatinib in GBM cells by targeting NDUFA4L2 in vivo, we subcutaneously injected LN229 cells into nude mice. As described previously, we selected 50 mg/kg apatinib and 3 mg/kg Mdivi-1 for in vivo experiments^12,23,28^. The results showed that there was no significant tumor suppression in the Mdivi-1-treated group, compared with the control group. Apatinib treatment, with or without Mdivi-1, resulted in significant tumor suppression in all nude mice. Moreover, apatinib + Mdivi-1 treatment resulted in a greater reduction in tumor volume, compared with apatinib or Mdivi-1 alone (Fig. 6A–C). Furthermore, we used immunohistochemical staining to evaluate the Ki67, LC3, cleaved-PARP, and cleaved-caspase 3 in the xenograft tumors; we observed that apatinib + Mdivi-1 treatment resulted in marked elevation of cleaved-PARP and cleaved-caspase 3 expression levels, compared with the other groups (Fig. 6D, E). A significantly greater percentage of TUNEL-positive cells was detected in the apatinib + Mdivi-1 treatment group, compared with the other groups (Fig. 6D, E). We also recorded the survival of mice (*n* = 8) in the different treatment groups. Compared with the control group, the median survival of tumor-bearing mice was significantly prolonged in the apatinib-treated group. Tumor-bearing mice treated with apatinib + Mdivi-1 had a significantly longer median survival than apatinib-treated mice (Fig. 6F). These findings strongly indicate that the mitophagy inhibitor Mdivi-1 can enhance the anti-tumor effect of apatinib in vivo.

## Discussion

Glioblastoma, the most common malignant tumor of the central nervous system, has a rapid course and extremely poor prognosis. Treatment options are limited for patients with GBM. As the standard treatment, Stupp’s regimen has been widely used for more than 10 years; however, the median survival of patients with GBM who are treated with this regimen is limited to 14.6 months^2,4,32,33^. Following standard treatment of patients with GBM, tumor recurrence and chemotherapy resistance remains a considerable challenge^34^. Therefore, the identification of new therapeutic targets and the development of novel treatment strategies are important.

The mitochondrion is an important intracellular organelle in eukaryotic cells; it is the major site of oxidative phosphorylation and generation of ATP, as well as byproducts of reactive oxygen species. The mitochondrial respiratory chain, also known as the electron transport chain, is composed of five respiratory complexes. NDUFA4L2, a subunit of Complex I of the mitochondrial respiratory chain, has an important role in metabolic reprogramming and oxidative stress in malignant tumors^7,35^. Several recent studies have revealed the roles of NDUFA4L2 in various tumors. NDUFA4L2 reportedly acts as an oncogene; notably, it is a direct transcriptional target of HIF-1α in hepatocellular carcinoma, clear cell renal cell carcinoma, lung cancer, and colorectal cancer^6,8,9^. However, the role of NDUFA4L2 in GBM and its related mechanism have been unknown. In GBM, the accumulation of HIF-1α often occurs under hypoxic conditions; under normoxia, expression of HIF-1α is very low or absent^36–38^. The present study is the first to show that NDUFA4L2 is highly upregulated in GBM tissues and cells; moreover, elevated NDUFA4L2 expression was identified as an independent prognostic marker for overall patient survival. Notably, NDUFA4L2 knockdown may arrest the cell cycle, induce apoptosis, and initiate protective mitophagy in GBM. Our results suggest that NDUFA4L2 acts as an oncogene; it may also serve as a prognostic biological marker and novel potential target in GBM. Based on a large sample of clinical data and our experimental results, we determined that the expression level of NDUFA4L2 is not directly regulated by HIF-1α in GBM, indicating that other mechanisms may regulate NDUFA4L2 in GBM. The HIF-1α inhibitors digoxin and 2-ME could not inhibit the expression of NDUFA4L2 in GBM, which may present challenges in the clinical application of NDUFA4L2 targeting in patients with GBM.

We also showed that apatinib can effectively target NDUFA4L2 in GBM. Apatinib efficiently inhibited the expression of NDUFA4L2, which caused GBM cell cycle arrest, induced apoptosis, and forced cells to initiate protective mitophagy in vitro and in vivo; notably, autophagy inhibition could significantly enhance the anti-cancer effects of apatinib. Apatinib, as a multikinase inhibitor, has been shown to exhibit good anti-cancer effects in various clinical trials^14,39,40^. But interestingly, the inhibitory effect on NDUFA4L2 is not available to all multikinase inhibitors, which we have made a preliminary exploration (Supplementary Fig. S4). Apatinib can maintain a high blood concentration in the body by oral or intravenous injection and can effectively penetrate the blood-brain barrier^41^; it is also well-tolerated in the body^42,43^. Thus, our results demonstrate that apatinib could be a potential therapeutic strategy for GBM by targeting NDUFA4L2.

## Supplementary information

Supplementary Figure S1–4

Supplementary Tables
