# Supplementary material for: NDUFA4L2 promotes glioblastoma progression, is associated with poor survival, and can be effectively targeted by apatinib
Source: Cell Death Dis. 2021 Apr 7;12(4):377. doi: 10.1038/s41419-021-03646-3 (PMC8027655; doi:10.1038/s41419-021-03646-3)
Supplement: Supplementary file 1 — Supplementary Figure S1–4 [file 41419_2021_3646_MOESM1_ESM.docx]

**Supplementary Figure S1-4**


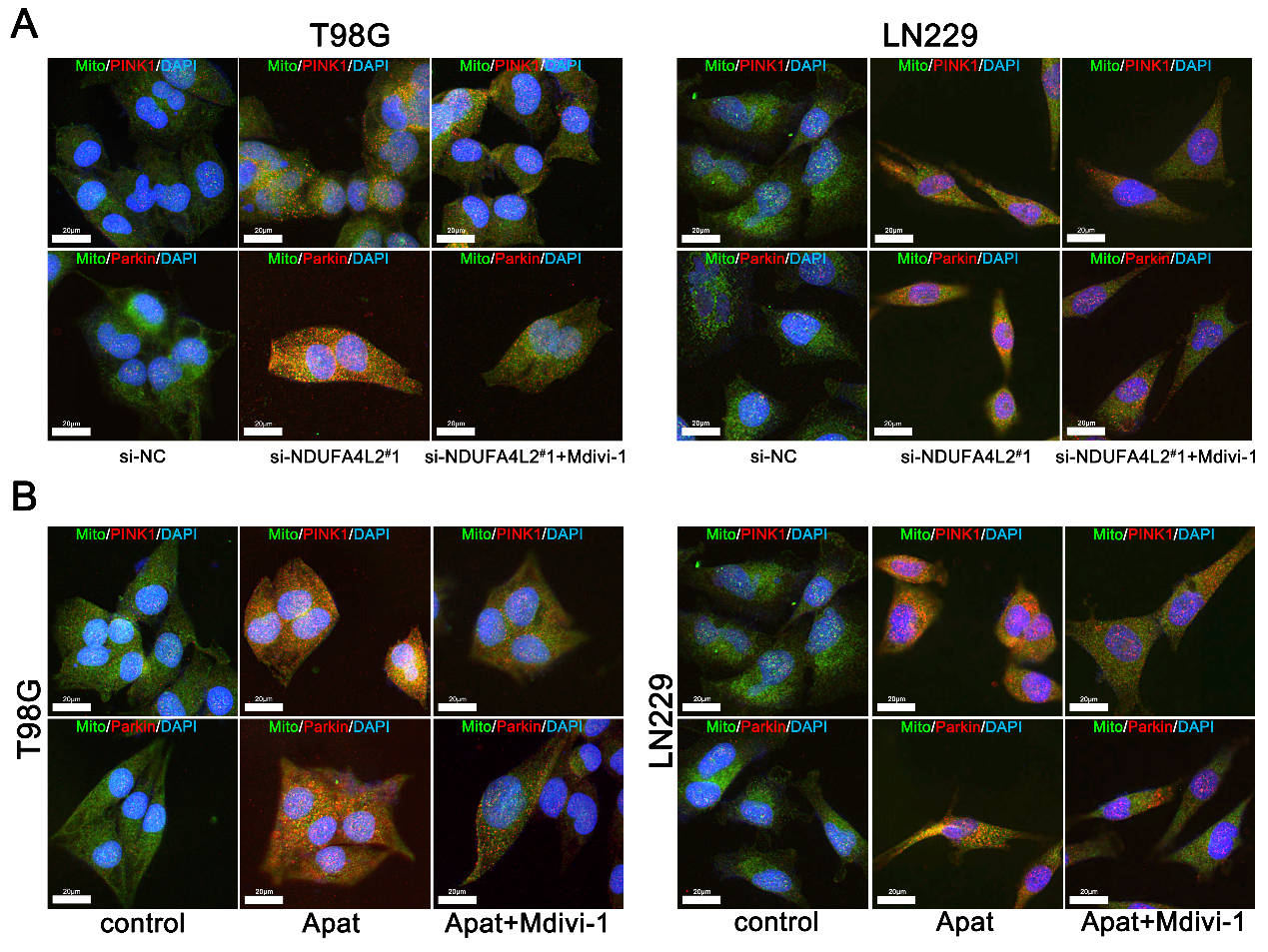


**Figure. S1 NDUFA4L2 knockdown or treated the cells with apatinib not only increased PINK1 and Parkin levels, but also promoted PINK1 and Parkin localization to mitochondria in GBM cells. And these changes can be reversed by Mdivi-1. A, B** T98G and LN229 cells were transfected with si-NC and si-NDUFA4L2#1 or treated with apatinib(40 μM) or Mdivi-1(5 μM) for 24h. Representative immunostaining images of 3 independent experiments demonstrated co-localization of PINK1 and Parkin with mitochondria in GBM cells.


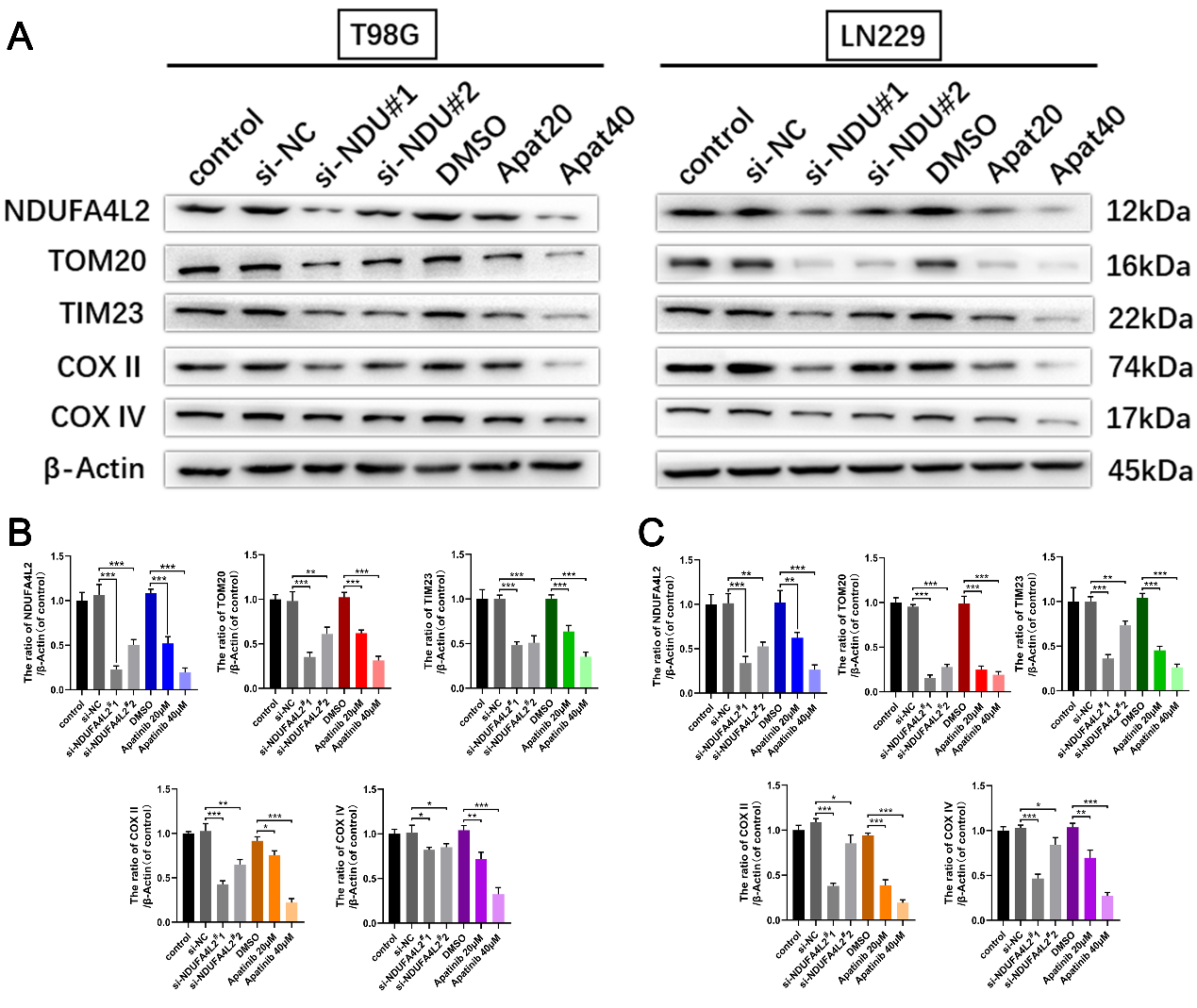


**Figure. S2 NDUFA4L2 knockdown or GBM cells were treated with apatinib induced the expression of some mitochondrial marker levels decreased uniformly. A** The expression of some mitochondrial marker (including COX II, COX IV, TOM20, TIM23) levels was detected in T98G and LN229 cells with NDUFA4L2 knockdown(si-NC, si-NDUFA4L2^#^1 and si-NDUFA4L2^#^2) and Apatinib (20, 40μM) treatment by western blot assays. **B, C** Quantitative analysis of the expression of protein (n = 3). **p* < 0.05, ***p*<0.01, ****p*<0.001 compared with control groups.


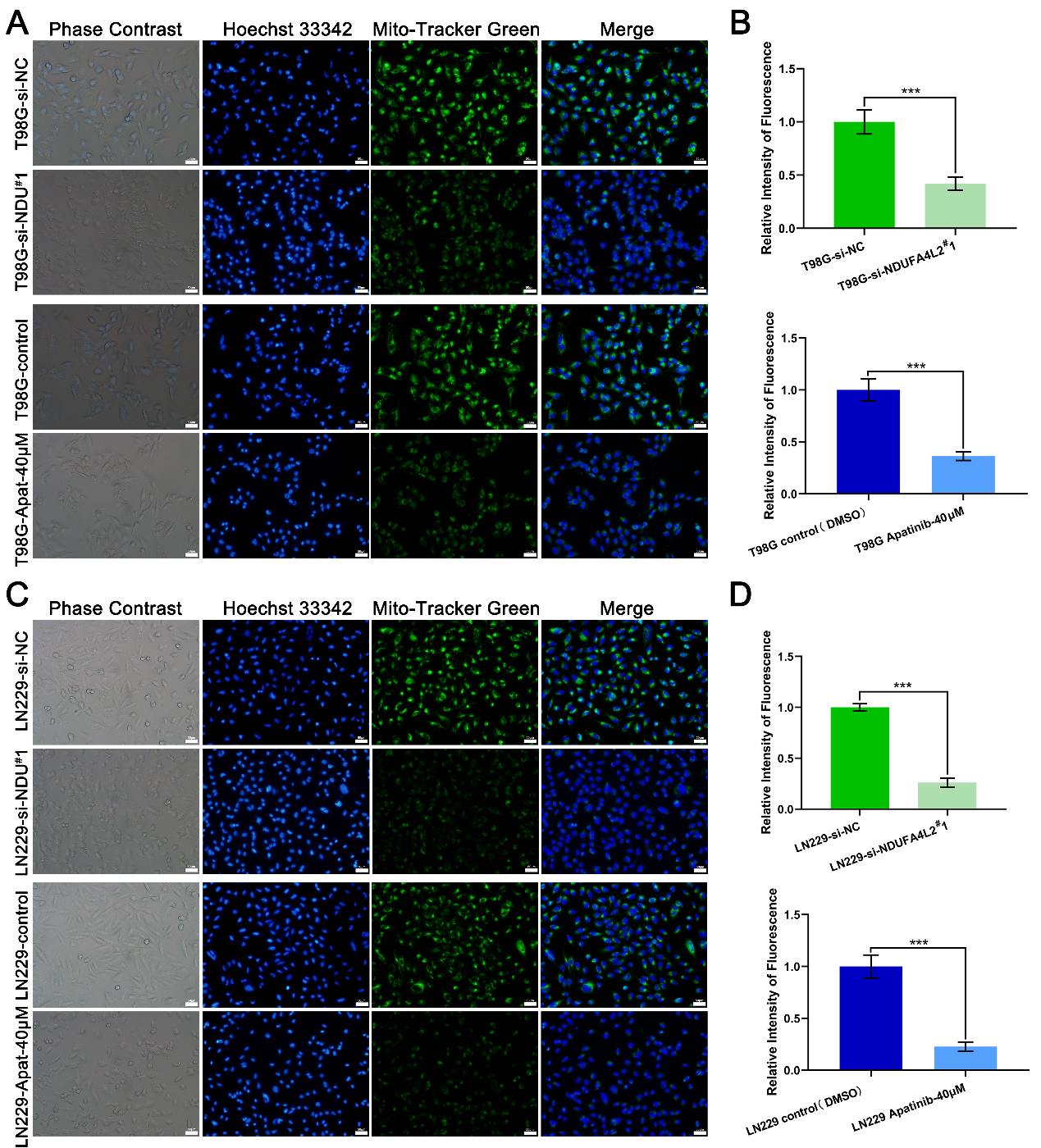


**Figure. S3 NDUFA4L2 knockdown or treated the cells with apatinib caused a significant decrease in the activity of intracellular mitochondria. A, C** Mitotracker probe was used to determine the mitochondrial mass by Mito-tarcker Green in T98G and LN229 cells. **B, D** Quantitative analysis of the intensity of fluorescence (n = 3). ****p*<0.001 compared with control groups.


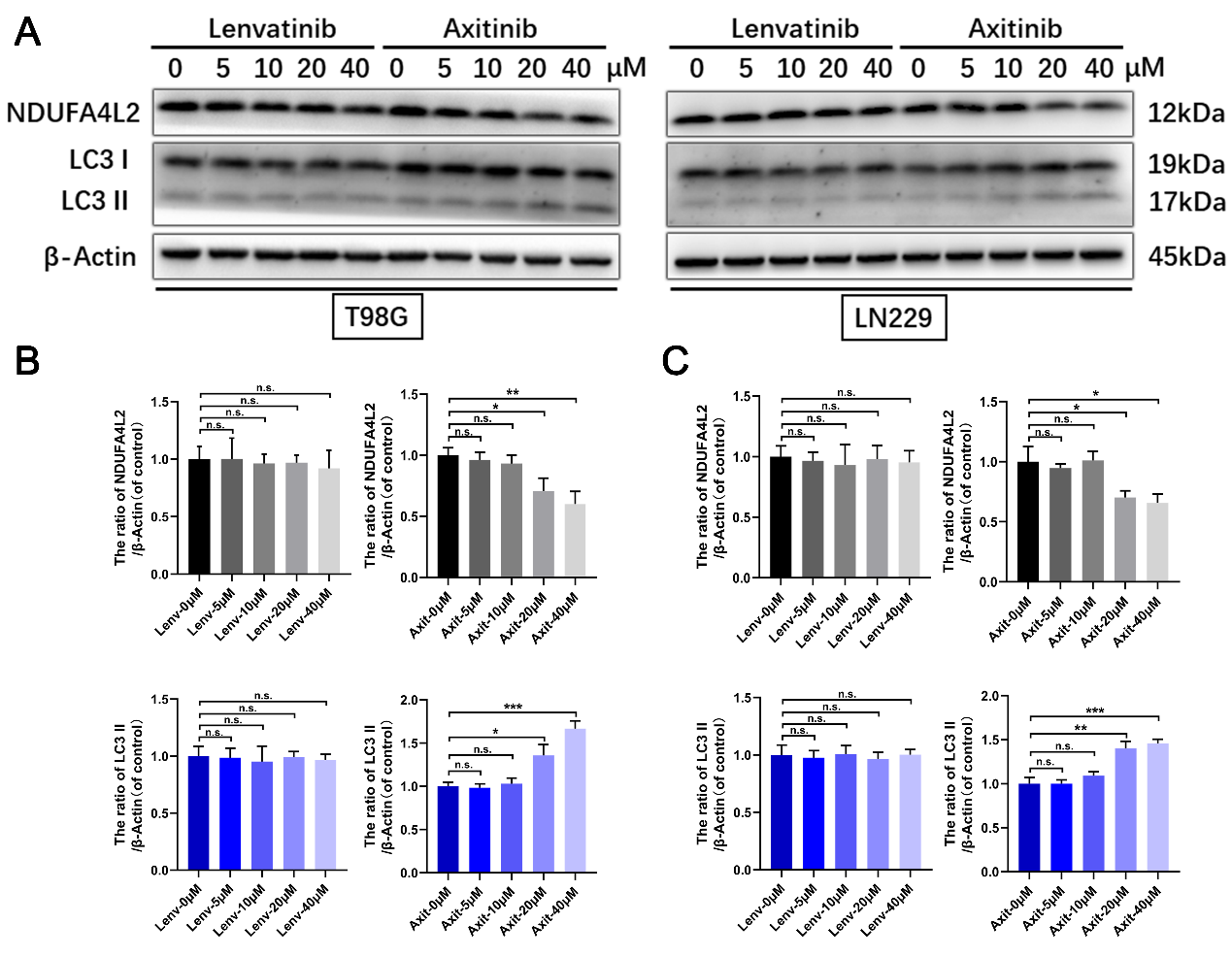


**Figure. S4 The inhibitory effect of Lenvatinib and Axitinib on the expression of NDUFA4L2 in glioblastoma cells. A-C.** The protein levels of NDUFA4L2 and LC3 in T98G and LN229 cells treated respectively by Lenvatinib and Axitinib with increasing concentration (0, 5, 10, 20 and 40μM) were quantified by western blot assays (n = 3). **p* < 0.05, ***p*<0.01, ****p*<0.001 compared with control groups. Lenv, Lenvatinib; Axit, Axitinib.
