# Supplementary material for: NDUFA4L2 promotes glioblastoma progression, is associated with poor survival, and can be effectively targeted by apatinib
Source: Cell Death Dis. 2021 Apr 7;12(4):377. doi: 10.1038/s41419-021-03646-3 (PMC8027655; doi:10.1038/s41419-021-03646-3)
Supplement: Supplementary file 2 — Supplementary Tables [file 41419_2021_3646_MOESM2_ESM.docx]

**Supplementary Tables.**

**Table S1. Reagents ­and­ chemicals** **Information**

| **Reagents ­and chemicals** | **Molecular formula** | **Molecular**  **weight** | **Purity** | **Source** |
| --- | --- | --- | --- | --- |
| Apatinib | C_25_H_27_N_5_O_4_S | 493.58g/mol | >98% | MedChemExpress,  State of New Jersey, USA |
| 2-Methoxyestradiol (2-Me) | C_19_H_21_O_3_ | 302.41g/mol | >98% | MedChemExpress,  State of New Jersey, USA |
| Mdivi-1 | C_15_H_10_C_l2_N_2_O_2_S | 353.22g/mol | >98% | Sigma-Aldrich,  City of Saint Louis, USA |
| Digoxin | C_41_H_64_O_14_ | 780.94g/mol | >98% | Sigma-Aldrich,  City of Saint Louis, USA |

**Table S2. Primer sequences used for qRT-PCR**

| **Gene** | **Primers** |  |
| --- | --- | --- |
| β-actin | Forward | 5′-AAAGACCTGTACGCCAACAC-3′ |
|  | Reverse | 5′-GTCATACTCCTGCTTGCTGAT-3′ |
| NDUFA4L2 | Forward | 5′-GCTTTACTTGCTGCGACTCG-3′ |
|  | Reverse | 5′-CCGGTCCTTCTTCAGCTTCTTA-3′ |

**Table S3. Antibodies information**

| **Antibody** | **Dellution ratio** | **Catalog#** | **Source** |
| --- | --- | --- | --- |
| NDUFA4L2 | 1:1,000 | Ab74138 | Abcam, Cambridge, MA |
| HIF-1α | 1: 500 | Ab51608 | Abcam, Cambridge, MA |
| ATG7 | 1:1,000 | Ab52472 | Abcam, Cambridge, MA |
| Beclin1 | 1:1,000 | Ab207612 | Abcam, Cambridge, MA |
| P62 | 1:1,000 | Ab109012 | Abcam, Cambridge, MA |
| PINK1 | 1:1,000 | Ab23707 | Abcam, Cambridge, MA |
| Parkin | 1:1,000 | Ab77924 | Abcam, Cambridge, MA |
| LC3 | 1:1,000 | NB100-2220 | Novus, Colorado, USA |
| cyclin D1 | 1:1,000 | 2922S | Cell Signaling, Danvers, MA |
| PARP | 1:1,000 | 9542S | Cell Signaling, Danvers, MA |
| caspase3 | 1:1,000 | 9662S | Cell Signaling, Danvers, MA |
| cleaved-caspase3 | 1:1,000 | 9664S | Cell Signaling, Danvers, MA |
| Bax | 1:1,000 | 2774S | Cell Signaling, Danvers, MA |
| Bcl2 | 1:1,000 | 15071S | Cell Signaling, Danvers, MA |
| Ki-67 | 1:1,000 | 9449S | Cell Signaling, Danvers, MA |
| β-actin | 1:1,000 | AA128 | Beyotime, Shanghai, China |
